# Prefoldin Function in Cellular Protein Homeostasis and Human Diseases

**DOI:** 10.3389/fcell.2021.816214

**Published:** 2022-01-17

**Authors:** Ismail Tahmaz, Somayeh Shahmoradi Ghahe, Ulrike Topf

**Affiliations:** Laboratory of Molecular Basis of Aging and Rejuvenation, Institute of Biochemistry and Biophysics, Polish Academy of Sciences, Warsaw, Poland

**Keywords:** prefoldin, molecular chaperone, proteostasis, TRIC, PFDN, neurodegenerative diseases, cancer

## Abstract

Cellular functions are largely performed by proteins. Defects in the production, folding, or removal of proteins from the cell lead to perturbations in cellular functions that can result in pathological conditions for the organism. In cells, molecular chaperones are part of a network of surveillance mechanisms that maintains a functional proteome. Chaperones are involved in the folding of newly synthesized polypeptides and assist in refolding misfolded proteins and guiding proteins for degradation. The present review focuses on the molecular co-chaperone prefoldin. Its canonical function in eukaryotes involves the transfer of newly synthesized polypeptides of cytoskeletal proteins to the tailless complex polypeptide 1 ring complex (TRiC/CCT) chaperonin which assists folding of the polypeptide chain in an energy-dependent manner. The canonical function of prefoldin is well established, but recent research suggests its broader function in the maintenance of protein homeostasis under physiological and pathological conditions. Interestingly, non-canonical functions were identified for the prefoldin complex and also for its individual subunits. We discuss the latest findings on the prefoldin complex and its subunits in the regulation of transcription and proteasome-dependent protein degradation and its role in neurological diseases, cancer, viral infections and rare anomalies.

## Introduction

Protein homeostasis (proteostasis) plays a vital role in various biological processes. Proteostasis is facilitated by the correct folding of proteins and removal of misfolded proteins to avoid protein aggregation, which can be toxic for the cell. To maintain proteostasis, cells contain various protein chaperones to check protein quality and conformation. A large family of chaperones, collectively called heat shock proteins (HSPs), are involved in the surveillance of misfolded and unfolded proteins in both the nucleus and cytosol (Hartl and Hayer-Hartl, 2002; Jones and Gardner, 2016). Non-native polypeptides must be sensed by chaperones and assisting proteins to assure correct folding. Disruption of the cooperation of chaperones and their assisting co-chaperones can result in protein misfolding and aggregation. Consequently, proteostasis can be impaired and drive the development of various pathologies (Hartl and Hayer-Hartl, 2002; Jones and Gardner, 2016).

Monomeric molecular chaperones are well established to assist in the folding of proteins into their native structure and responsible for the remodeling of proteins with erroneous conformations (Saibil, 2013). In addition to monomeric molecular chaperones, oligomeric chaperonins contribute to protein folding. Chaperonins can be divided into two groups. Group I chaperonins are found in bacteria and organelles of endosymbiotic origin (e.g., chloroplasts and mitochondria). Group II chaperonins exist in eukaryotes and archaea (Russmann et al., 2012). One well-known member of Group II chaperonin is tailless complex polypeptide 1 ring complex (TRiC)/chaperonin containing tailless complex polypeptide 1 (CCT). TRiC/CCT localizes to both the nucleus and cytosol (Marracci et al., 2015).

Unlike prokaryotes, eukaryotes and archaea have another chaperone-assisted protein (Sahlan et al., 2018). Prefoldin is a co-chaperone of TRiC/CCT (Millan-Zambrano and Chavez, 2014; Zhang J. et al., 2020). It is a heterohexameric protein complex (Glover and Clark, 2015) that is composed of two α subunits (PFD3 and PFD5) and four β subunits [PFD1, PFD2, PFD4, and PFD6; (Siegert et al., 2000; Whitehead et al., 2007). All subunits differ in their amino acid sequence. In contrast, archaea prefoldin complex consists of six subunits composed of two identical α and four identical β subunits (Sahlan et al., 2018). Although prefoldin was not found in prokaryotes, its genetic origin is similar to chaperones that were found in prokaryotes (Bogumil et al., 2014). Likewise, the prefoldin complex is evolutionarily conserved in higher eukaryotes, including humans and plants (Siegert et al., 2000; Cao, 2016).

**FIGURE 1:**
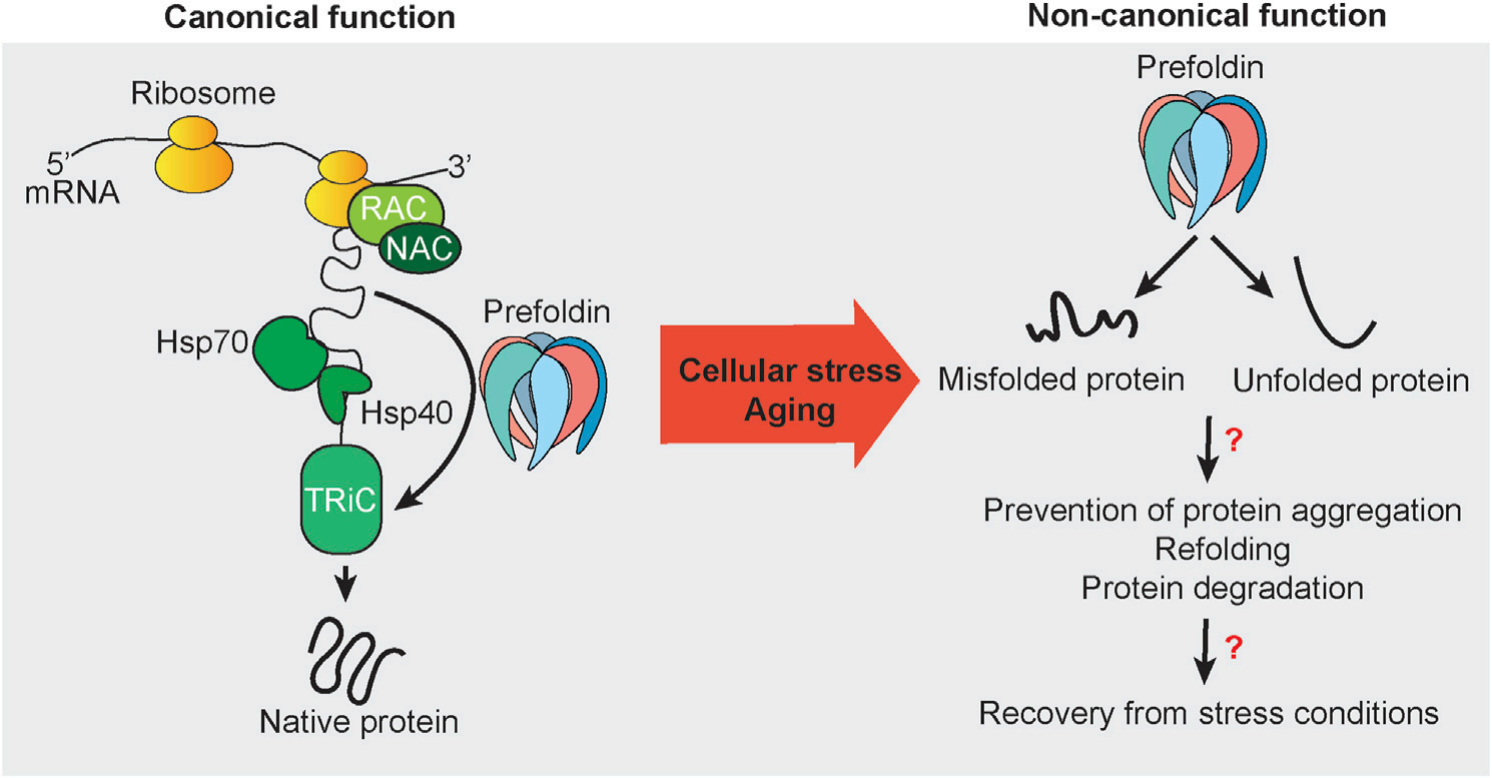
Canonical and non-canonical functions of prefoldin. Nascent chains that emerge from actively translating ribosomes are subjected to protein folding that is mediated by the protein chaperones RAC, NAC, Hsp70, Hsp40, and TRiC. Some newly synthesized proteins utilize the co-chaperone prefoldin to guide substrates directly to its downstream folding chaperone TRiC. The canonical function of prefoldin results in substrate proteins with the native fold. Under stress conditions and potentially during aging, prefoldin is associated with misfolded and unfolded proteins. Mostly unknown are the nature of such cellular substrates *in vivo* and cellular consequences and mechanisms that may be necessary to recover from cellular stress conditions to restore cellular homeostasis.

The prefoldin complex assists in the folding of newly synthesized polypeptides that are produced on ribosomes (Figure 1). In archaea, prefoldin exerts this function in combination with group II chaperonin. In eukaryotes, the canonical function of prefoldin is thought to be more specialized. In eukaryotes, prefoldin transfers mainly the cytoskeletal proteins actin and tubulin to TRiC/CCT (Abe et al., 2013; Povarova et al., 2014; Zhang et al., 2016). This transfer is also supported by cooperation with the monomeric chaperones HSP40 and HSP70 in a co-translational manner (Millan-Zambrano and Chavez, 2014; Gestaut et al., 2019) and required for accelerating the maturation of cytoskeletal proteins (Gestaut et al., 2019). During this process, nascent polypeptide chains bind to prefoldin in the initial phase (Kabir et al., 2011) and are transferred to TRiC/CCT in late stages of protein folding (Comyn et al., 2016; Gestaut et al., 2019). Binding of prefoldin subunits with TRiC/CCT subunits decreases the association of nascent chain with prefoldin (Gestaut et al., 2019; Liang et al., 2020). Thus, nascent chains are released from prefoldin and folded in an adenosine triphosphate (ATP)-dependent manner by TRiC/CCT. The holdase function of prefoldin is ATP-independent, similar to stress-inducible small HSPs (Webster et al., 2019). Its ATP independence raises the question of whether prefoldin has additional functions under conditions of low cellular energy levels such as mitochondrial dysfunction and metabolic disorders, aging, or chronic stress (Chaudhari and Kipreos, 2018; Andreasson et al., 2019; Fang et al., 2021).

Actin comprises 5–10% of cellular protein mass (Cooper, 2000). Thus, prefoldin is thought to be abundant with constitutive expression. However, the regulation of prefoldin expression has been scarcely addressed in the literature. The turnover of prefoldin subunits is regulated by ubiquitin-dependent degradation. However, prefoldin can become resistant to ubiquitination by forming a β-hairpin and coil-coil structure (Liang et al., 2020). Since the discovery of prefoldin in 1998 (Vainberg et al., 1998), much research has been performed to elucidate the prefoldin structure (Hansen et al., 1999) and describe its canonical functions as a co-chaperone (Sahlan et al., 2018). However, recent research has indicated involvement of the prefoldin complex and its subunits in withstanding cellular stress and maintaining protein homeostasis. Research on the non-canonical function of prefoldin is still scattered, consisting of the regulation of transcription, the regulation of protein aggregate formation, and protein degradation (Figure 1).

Alongside fundamental research describing various functions of prefoldin it is suggested that prefoldin is a potential biomarker and therapeutic target for human diseases that are associated with the disruption of protein homeostasis. The present review discusses non-canonical functions of prefoldin in eukaryotes and the involvement of prefoldin in human diseases.

## Non-Canonical Functions of the Prefoldin Complex

Apart from the canonical function of the cytosolic prefoldin complex as a co-chaperone for the co-translational folding of cytoskeletal proteins, all prefoldin subunits were found to have unique and specific interactors. These interactions presumably allow prefoldin subunits to engage in non-canonical cellular functions (Simons et al., 2004), but describing all of these interactions is beyond the scope of this review. Table 1 summarizes the identified interactors with different prefoldin subunits. A recent review described links between interactors of prefoldin subunits and the progression of cancer (Liang et al., 2020; Herranz-Montoya et al., 2021). The sections below focus on the involvement of prefoldin subunits in transcription, protein aggregation, and degradation.

**TABLE 1:** Interactions between prefoldin subunits and other proteins.

Prefoldin subunit	Interactor	Organism	Function	References
PFDN1	HLA-G	Human	Progression of pregnancy	Liu et al. (2013)
	cyclin A	Human	Cancer progression	Wang et al. (2017)
	RABV	Mouse	Upregulation in virus infection	Zhang et al. (2015)
	FILIP1L	Mouse and human	Progression of mucinous colon cancer	Kwon et al. (2021)
PFD2/PFDN2	Irc15	Yeast	Sister chromatid cohesion	Ming Sun et al. (2020)
	γ-synuclein	Mouse	Expression regulation in neurodegenerative disease	Chintalapudi and Jablonski, (2017)
	DELLA	Populus	Alteration of lignin content	Zhang W et al., (2020)
PFD3	pVHL	Human	Tumor suppression	Kim et al. (2018)
	HDAC1	Human	Delivery to CCT complex	Banks et al. (2018)
	hMSH4	Human	Degradation	Xu and Her, (2013)
	DELLA	A. thaliana	Stress-dependent interaction	Locascio et al. (2013)
PFD4	Irc15	Yeast	Sister chromatid cohesion	Ming Sun et al. (2020)
	LSM8	A. thaliana	Splicing	Esteve-Bruna et al. (2020)
PFDN5	c-Myc	Human	Inhibitory effect on oncogenes	Satou et al. (2001)
	TIF1β	Human	Inhibitory effect on oncogenes	Hagio et al. (2006)
	Rabring7	Human	c-Myc degradation	Narita et al. (2012)
	Egr1	Human	c-Myc inhibition	Yoshida et al. (2008)
	ARFP/F	Human	c-Myc activation	Ma et al. (2008)
	p73α	Human	c-Myc inhibition	Watanabe et al. (2002)
	p63α	Human	c-Myc activation	Han et al. (2016)
	ΔNp63α	Human	Senescence	Chen et al. (2018)
	Fabp4	Mouse	Regulation of sperm differentiation at transcription	Yamane et al. (2015)
	PLa2g3	Mouse	Transcriptional regulation of sperm number and motility	Yamane et al. (2015)
	PLa2g10	Mouse	Transcriptional regulation of sperm number and motility	Yamane et al. (2015)
	DELLA	A. thaliana	Stress-dependent interaction	Locascio et al. (2013)
PFD6/PFD-6	DELLA	A. thaliana	Stress-dependent interaction	Locascio et al. (2013)
	FOXO	C. elegans	Transcriptional regulation of longevity	Son et al. (2018)
	Irc15	Yeast	Sister chromatid cohesion	Ming Sun et al. (2020)
All subunits	mst	Drosophila	Assembly of spindle microtubules	Palumbo et al. (2015)

### Regulation of Transcription

**FIGURE 2:**
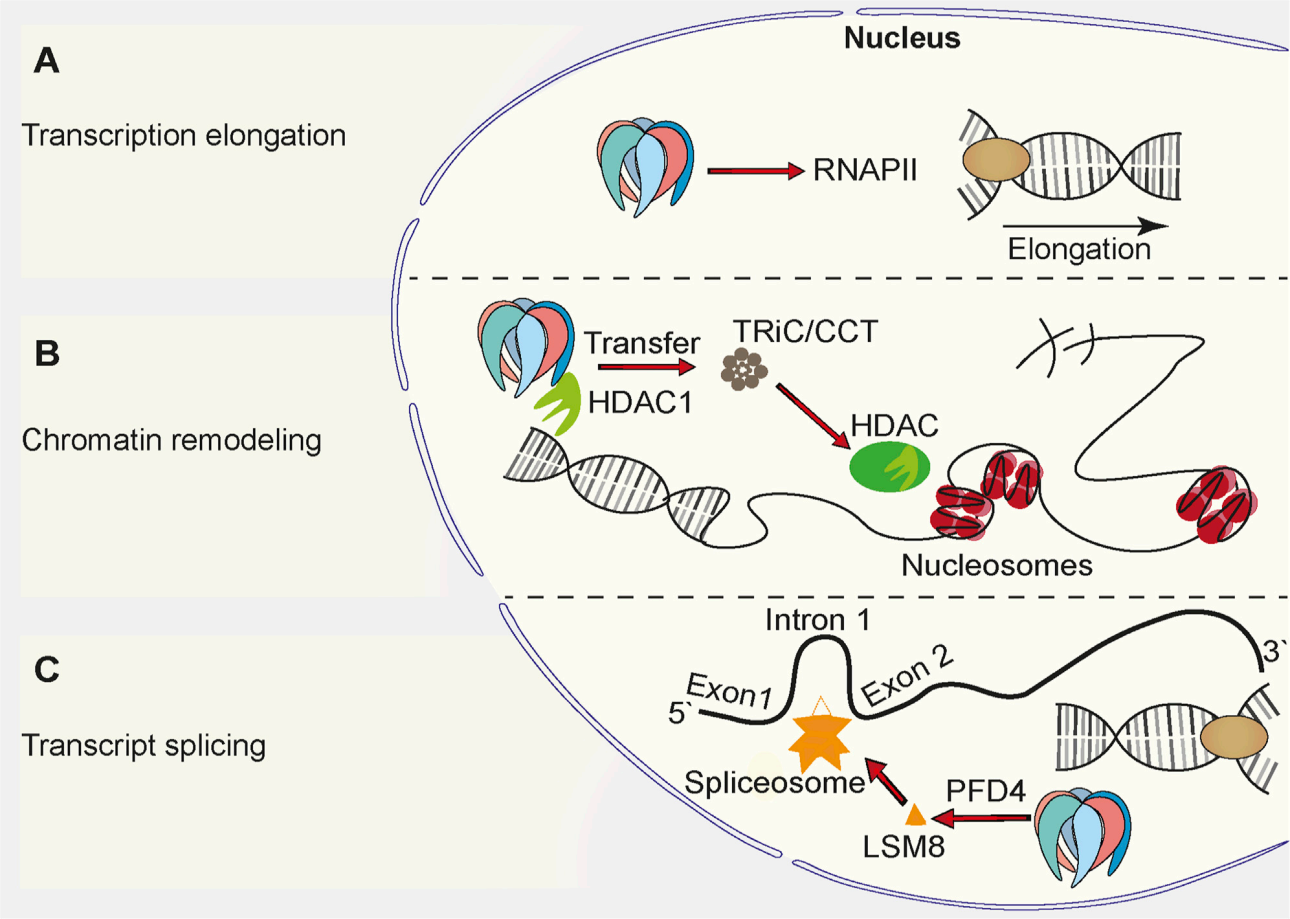
Function of prefoldin complex in the cell nucleus. **(A)** Prefoldin stimulates transcription elongation by promoting binding of RNA Polymerase II (RNAPII) to euchromatin. **(B)** Prefoldin interacts with HDAC1 and supports incorporation of it into its active complex via TRiC/CCT complex. The interaction favors chromatin remodeling. **(C)** The PFD4 subunit is required for the incorporation of the spliceosome core complex subunit, LSM8, into the spliceosome.

Prefoldin was described to be a cytoplasmic protein complex (Geissler et al., 1998). The deletion of *PFD1* in yeast resulted in unexpected defects in transcription elongation (Millan-Zambrano et al., 2013), but these findings complemented other studies and suggested a role for prefoldin in the nucleus (Millan-Zambrano and Chavez, 2014; Mousnier et al., 2007; Watanabe et al., 2002) (Figure 2). All prefoldin subunits were found to exhibit nucleo-cytoplasmic localization in the yeast *Saccharomyces cerevisiae* (Millan-Zambrano et al., 2013). This newly identified transcriptional function of prefoldin subunits was independent of actin assembly in the cytoplasm and independent of the involvement of nuclear actin in transcription elongation. The deletion of *PFD1* decreased the RNA polymerase II occupancy of gene bodies (Figure 2A). This was explained by the role of prefoldin in the removal of histones from chromatin. These data were supported by numerous genetic interactions between prefoldin subunits and chromatin factors (Millan-Zambrano et al., 2013; Ming Sun et al., 2020). Genetic interactions between prefoldin and TRiC/CCT components and chromatin factors were similar and suggested a combined action of chaperonin and its co-chaperone in transcription regulation in the nucleus. Furthermore, the physical interaction between histone deacetylase 1 (HDAC1) and prefoldin and TRiC/CCT suggested the requirement for chaperone-mediated folding before its assembly into the active complex (Banks et al., 2018) (Figure 2B). This further strengthened the involvement of prefoldin in the remodeling of chromatin and transcription regulation. A recent study in *Arabidopsis* showed that PFD4 mediates the chaperone-client interaction between Hsp90 and LSM8, a central component of the spliceosome core complex. Prefoldin was shown to be important for maintaining adequate levels of the spliceosome core complex (Esteve-Bruna et al., 2020) (Figure 2C). A strong genetic interaction between *pfd4*Δ (gim3 in yeast) and *lsm8*Δ in yeast (Costanzo et al., 2016) prompted Esteve-Bruna *et al* to the notion that the functional interaction between prefoldin and the spliceosome could be conserved. The implication of prefoldin in the regulation of co-translational splicing effects was also recently demonstrated for human prefoldin (Payán-Bravo et al., 2021). The binding of prefoldin to transcribed genes was detected genome-wide. The loss of prefoldin function led to a decrease in the phosphorylation of elongating polymerase II and decrease in the binding of splicing factors to chromatin. Links between prefoldin and cellular splicing machinery thus add another level of complexity to the ways in which prefoldin influences transcriptional processes in the nucleus.

### Role in Cellular Stress Defense

**FIGURE 3:**
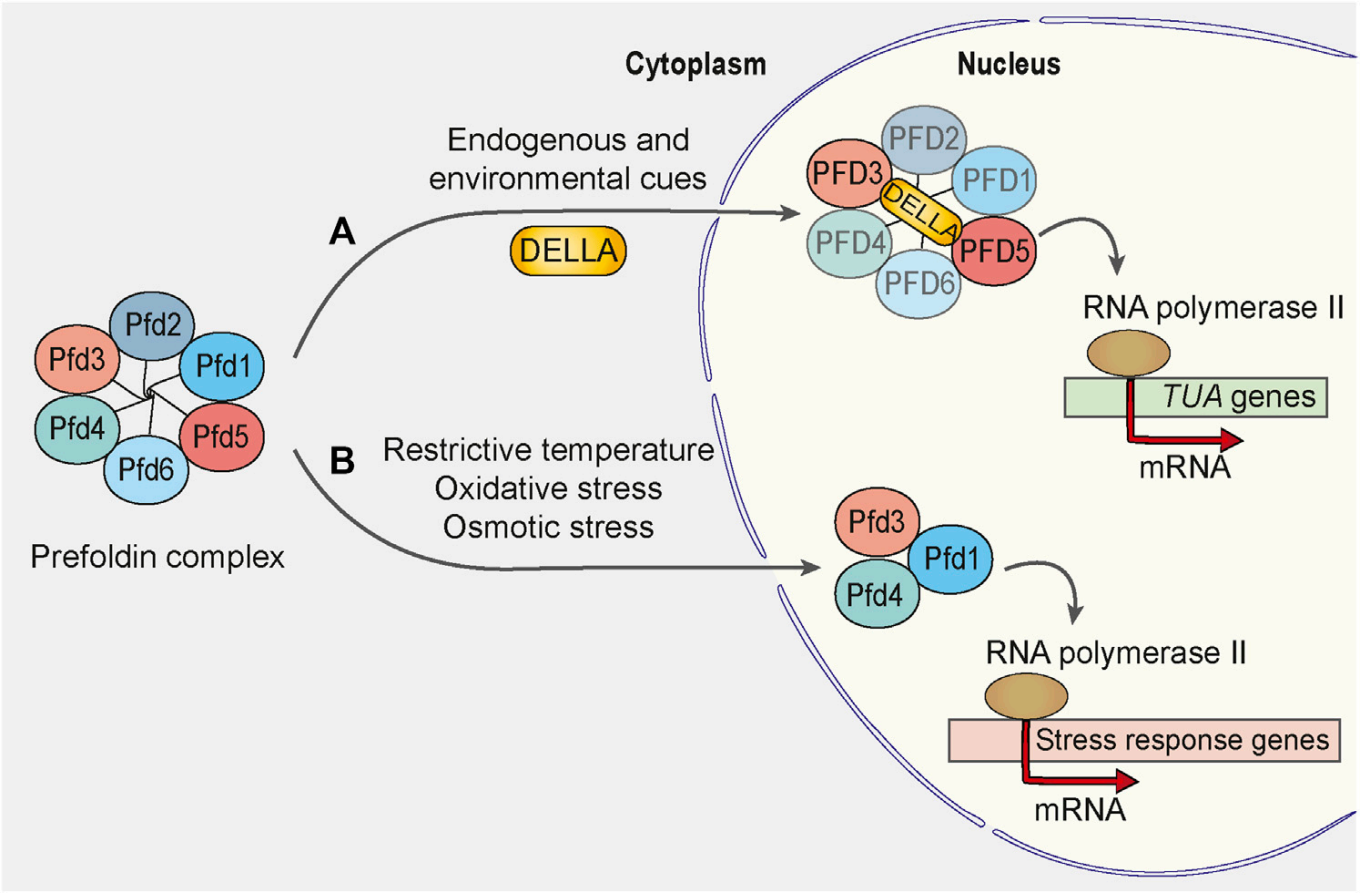
Prefoldin complex is involved in transcriptional regulation upon cellular stress. **(A)** In *Arabidopsis*, endogenous and environmental stimulators control the growth rate of a plant by inducing the DELLA-mediated accumulation of prefoldin complex in the cell nucleus. PFD3 and PFD5 interact directly with DELLA, but integrity of the prefoldin complex during the translocation remains unaffected. In the nucleus, prefoldin complex or some of its subunits activate the transcription of α-tubulin encoding genes (*TUA*). **(B)** In response to exposure of yeast *Saccharomyces cerevisiae* to stress stimuli such as restrictive temperature, oxidative and osmotic stress, Pfd1, Pfd3 and Pfd4 subunits localize to the nucleus to facilitate the transcription elongation of some stress-response genes. Pfd1, Pfd3 and Pfd4 may form a distinct complex from the hexameric prefoldin complex because the absence of other prefoldin subunits and consequent disruption of the prefoldin complex does not suppress the stress response in *S. cerevisiae*.

The importance of prefoldin-dependent regulation of gene expression became eminent under cellular stress conditions. The green fluorescent protein-tagged Pfd1 exhibited an increase in nuclear staining when cells were shifted to a restrictive growth temperature (Millan-Zambrano et al., 2013). This finding in yeast was consistent with studies in plants that investigated the regulated migration of prefoldin from the cytoplasm to the nucleus (Locascio et al., 2013). Here, the shuttling of plant prefoldin complex depended on the interaction of PFD3 and PFD5 with the transcriptional regulator DELLA under conditions of environmental and endogenous cue exposure (Figure 3A). A mutant of *Arabidopsis* that harbored deletions of all six prefoldin subunits was viable, and loss of the prefoldin complex did not significantly alter plant development under normal growth conditions. The prefoldin complex knockout mutant performed better than the wild type under osmotic stress conditions (Blanco-Touriñán et al., 2021). This indicates that prefoldin is needed to respond to stress conditions and/or environmental changes. Consistent with recent findings in plants are findings in yeast, in which Pfd3 (also called Gim2 or Pac10), Pfd4 (also called Gim3), and Pfd1 (also called Gim6) were shown to be involved in oxidative and osmotic stress-activated transcription (Amorim et al., 2017) (Figure 3B).

The deletion of prefoldin also affected yeast viability upon exposure to other exogenous stressors. A genome-wide screen of haploid deletion mutants found that subunits of the prefoldin complex were among the most sensitive mutants to arsenite treatment (Pan et al., 2010). Exposure to high temperatures decreased the growth of a *PFD5* deletion mutant in yeast (Gestaut et al., 2019). The induction of endoplasmic reticulum stress by thapsigargin treatment decreased cell viability in the absence of the prefoldin complex in mammalian cells (Abe et al., 2013). These stressors generally increase proteostatic stress in the cell, which might be an indication that prefoldin is required for the maintenance of protein homeostasis under conditions of high protein burden.

### Influence on Protein Turnover and Quality Control

Protein turnover defines the balance between protein production and protein degradation. Importantly, the correct folding of newly synthesized polypeptides is a limiting factor of both fundamental cellular processes (Rothman, 2010). Defects in the folding process can stall translation and require an increase in the removal of unfolded or misfolded proteins from the cell (Goldman et al., 2015). Nascent chains that emerge from the actively translating ribosome are passed to chaperones of the ribosome-associated complex (RAC) and nascent polypeptide-associated complex (NAC), which assist the folding of ∼70% of polypeptides (Balchin et al., 2016). Downstream of ribosomes (i.e., the hub of the chaperone network), Hsp70, together with Hsp40s, folds ∼20% of the proteome (Balchin et al., 2016). Ten percent of these proteins are passed further downstream to multi-domain cylindrical complexes, group II chaperonins [TRiC/CCT; Hsp60 chaperones in archaea and the eukaryotic cytosol (Thulasiraman et al., 1999; Yam et al., 2008);]. Some proteins bypass Hsp70 and are directly transferred to chaperonins via the prefoldin chaperone complex (Balchin et al., 2016) (see also Figure 1). The prefoldin structure was determined in archaea, revealing a jellyfish-shaped complex where the six subunits form six long coiled coils that are attached to a base of β barrels (Leroux et al., 1999; Fandrich et al., 2000; Siegert et al., 2000; Ohtaki et al., 2008). Biochemical experiments and low-resolution electron microscopy studies confirmed this structural arrangement for human prefoldin (Martin-Benito et al., 2002; Simons et al., 2004; Aikawa et al., 2015; Gestaut et al., 2019). In eukaryotes, charged residues in the distal end of coiled coils of certain prefoldin subunits bind to actin and tubulin, and defined residues in each of these substrates are necessary for this interaction. Thus, prefoldin binding is specific and may involve the combinatorial binding of different subunits for different targets. However, in contrast to archaeal prefoldin, which was shown to interact with numerous non-native substrates (Arranz et al., 2018), knowledge of the substrate landscape in eukaryotic prefoldin is limited. Interestingly, genome-wide genetic interaction screens in yeast revealed a large variety of genes that when deleted in the background of deletions of single prefoldin subunits result in a synthetic lethal phenotype (Goehring et al., 2003; Krogan et al., 2003; Tong et al., 2004; Friesen et al., 2006; Mitchell et al., 2008). This might indicate that prefoldin has a broader cellular function than previously anticipated.

**FIGURE 4:**
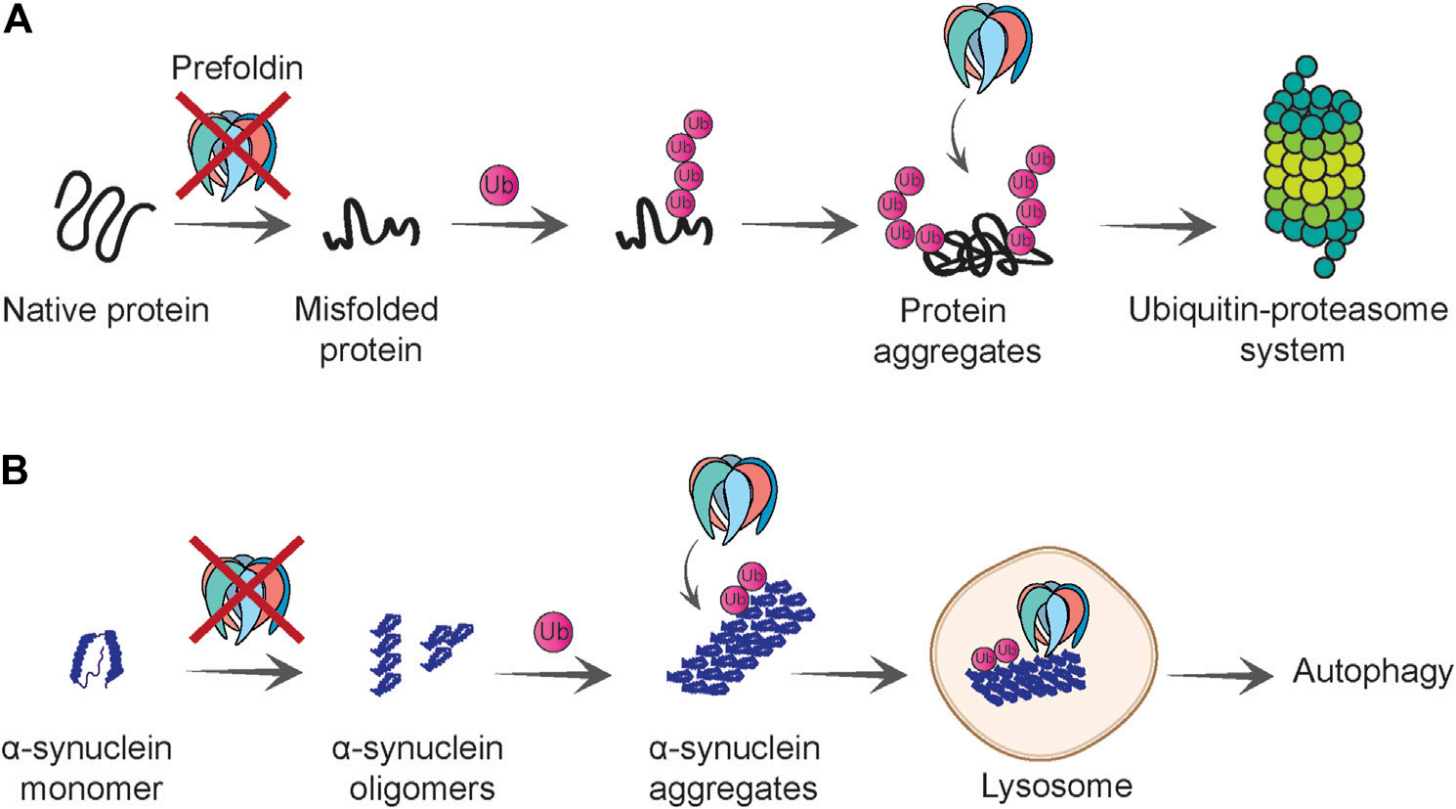
Role of prefoldin complex in protein quality control and degradation. **(A)** Prefoldin is required for polypeptides to gain native fold and loss of prefoldin function causes protein misfolding. Misfolded proteins that cannot be refolded are polyubiquitinated. Prefoldin complex co-localizes with the polyubiquitinated proteins and presumably facilitates their degradation by the ubiquitin-proteasome system. **(B)** Prefoldin complex prevents aggregation of α-synuclein at initial steps of oligomerization. If α-synuclein oligomers form aggregates, they will be ubiquitinated and in proximity to prefoldin complex delivered to the lysosome in which aggregates can be degraded in an autophagy-dependent manner.

A few findings suggest a link between the prefoldin complex and the removal of proteins that are targeted to the proteasome (Abe et al., 2013; Comyn et al., 2016) (Figure 4A). In a mammalian cell culture model, the prefoldin complex co-localized with ubiquitinated proteins that were found in aggregates upon proteasome inhibition. Intriguingly, lower levels of the prefoldin complex resulted in the higher accumulation of poly-ubiquitinated proteins that were resistant to detergent. This effect depended on concomitant inhibition of the proteasome. A decrease in prefoldin function that was associated with an insufficient protein degradation system most likely overwhelmed the cellular proteostasis system, consequently leading to an increase in cell death (Abe et al., 2013).

Overexpression of the prefoldin complex lowered levels of detergent-insoluble ubiquitinated protein species upon proteasome inhibition, but this conferred only a minimal beneficial effect on cell viability. A mouse model that expressed PFDN5 with a point mutation (MM-1α/PFDN5) showed normal PFDN5 expression and no detectable differences in expression of the prefoldin complex (Lee et al., 2011). However, cells that were derived from this MM-1α/PFDN5 mouse model accumulated more poly-ubiquitinated proteins and were more sensitive to proteasome inhibition. Superficially, no defect in the prefoldin complex was observed, but amino acid substitution in PFDN5 changed the structure of the subunit and altered its affinity for substrates (Abe et al., 2013). These findings demonstrated that the prefoldin complex is an important component of the protein quality control system and performs functions beyond simply acting as a co-translational holdase of newly synthesized polypeptides of cytoskeletal proteins. However, the mechanistic role of the prefoldin complex with regard to the proteasome remained unclear.

Revealing the extended landscape of prefoldin substrates in eukaryotes could shed light on the function of prefoldin during stress conditions, such as limited availability of the protein degradation system. To identify factors that promote the proteasomal degradation of misfolded proteins in yeast, the function of Pfd4 was characterized (Comyn et al., 2016). In the absence of *PFD4*, the model protein Guk1-7, which is thermally unstable and is a substrate of the proteasome, were partially protected from degradation. The inhibition of Guk1-7 turnover occurred independently of functionality of the proteasome complex and its ubiquitination levels. Importantly, Pfd4 was shown to interact with the mutant form of Guk1 but not the wildtype version. Thus, Pfd4 acts as a holdase for Guk1-7 to ensure its solubility. In the absence of *PFD4*, Guk1-7 localized in distinctive foci that were also positive for the aggregation markers Hsp42 and Hsp104. Other thermosensitive alleles were also tested. Different prefoldin subunits stabilized various model proteins to different extents. This indicates that each prefoldin subunit has differential affinity for their substrates (Comyn et al., 2016). This work opens the field for further exploration of a wider range of substrates. Much more work is needed to decipher interactions between single prefoldin subunits and specific substrates and substrate classes. Moreover, remaining unclear are whether the function of this holdase depends on a functional prefoldin complex or whether individual subunits act independently from their incorporation in the complex.

The involvement of the prefoldin complex and individual prefoldin subunits in protein quality control seems convincing, but unclear are how its function changes from being a canonical co-translational holdase to a translation-independent chaperone with holdase and disaggregase activity and whether this non-canonical function is independent of TRiC/CCT. Inhibition of the proteasome results in lower levels of amino acids, which in turn activates the integrated stress response. This universal response to unfavorable environmental conditions results in the attenuation of global translation by phosphorylation of eukaryotic initiation factor 2α (eIF2α) (Suraweera et al., 2012). Under such cellular conditions, co-translational chaperones might be free to perform other cellular tasks. Such a scenario was described for the ribosome-associated NAC and RAC-Ssb chaperone systems (Olzscha et al., 2011; von Plehwe et al., 2009; Wang et al., 2009).

### Implication in the Amelioration of Disease-Associated Protein Aggregation

Given the emerging role of prefoldin as a factor in maintaining protein solubility under physiological and cellular stress conditions, consistent observations have been made in model systems in which prefoldin can protect from toxic aggregates that are linked to human diseases.

The knockdown of PFDN2 in undifferentiated neuronal cells increased the formation of aggregates of polyQ stretches and polyQ-expanded huntingtin (HTT), gene defects that are related to the neurodegenerative Huntington’s disease (Tashiro et al., 2013). The knockdown of PFDN5 also affected aggregation but to a much lesser extent. Importantly, only the aggregation of pathogenic forms was enhanced and not healthy forms. The knockdown of PFDN2 or PFDN5 did not influence the expression of other chaperones, such as HSP70, HSC70, HSP40, and CCTα, suggesting that prefoldin could be a limiting factor to prevent aggregate formation. The overexpression of prefoldin reduced the aggregated form of pathogenic huntingtin. *In vitro* single-molecule observation by total internal reflection fluorescence microscopy showed that prefoldin suppressed the dimer-to-tetramer stage of HTT aggregation. This indicates that prefoldin inhibits the elongation stage of large oligomers of pathogenic HTT rather than inclusion formation *per se* (Tashiro et al., 2013).

Similar to HTT aggregation, the formation of α-synuclein aggregates increased upon the knockdown of prefoldin expression in a neuronal cell culture model (Takano et al., 2014). Moreover, ubiquitinated forms of fluorescently tagged wildtype and mutant α-synuclein co-localized with prefoldin (Figure 4B). This observation is consistent with the finding that prefoldin co-localizes with poly-ubiquitinated protein species (Abe et al., 2013). The co-localization of α-synuclein with prefoldin was detected in the lysosome, an organelle that can mediate α-synuclein clearance under normal conditions but also under conditions of high protein burden (Mak et al., 2010). Notably, only a small percentage of prefoldin was detected in the lysosome, whereas most remained in the cytoplasm. One unanswered question is whether the effect of prefoldin knockdown on α-synuclein aggregation and toxicity is a direct effect or a consequence of exhaustion of overall folding capacity of the cell. In either case, this work demonstrates that prefoldin might assist with the transfer of misfolded proteins to the autophagy-dependent degradation pathway (Takano et al., 2014) (Figure 4B).

The effect of prefoldin on the oligomerization of amyloid-β (Aβ) peptides was also investigated. The fibrillation of Aβ was inhibited in the presence of archaeal prefoldin (Sakono et al., 2008). A similar observation was made in an *in vitro* model (Sorgjerd et al., 2013). Deposits of Aβ are found in the brain cortex and hippocampus. Prefoldin was detected in both brain regions, but its expression was elevated in a mouse model that exhibited an increase in Aβ deposition. Amyloid-β cytotoxicity was reduced in a cell culture model when Aβ oligomers were formed in the presence of human prefoldin. However, the addition of recombinant human prefoldin did not prevent the toxic effect of preformed toxic Aβ oligomers lending further weight to the hypothesis that prefoldin acts on pre-oligomeric misfolded species (Sorgjerd et al., 2013).

In contrast to higher prefoldin expression in brain regions that exhibit an increase in Aβ deposition, a mouse model that expressed a mutant version of Tau exhibited a decrease in PFDN5 expression, which was age-dependent (Kadoyama et al., 2019).

Little is known about prefoldin function during biological aging. However, considering accumulating evidence that prefoldin function can have beneficial effects on the aggregation and toxicity of proteins that are linked to age-associated diseases, further efforts need to be made to elucidate the potential role of prefoldin during aging. The nematode *Caenorhabditis elegans* was proven to be a useful model organism for understanding the biology of aging. In *C. elegans*, prefoldin is essential in early development of the embryo because of the need for cytoskeletal structures (Lundin et al., 2008). The use of RNA interference (RNAi) technology allows the knockdown of genes in *C. elegans*. RNAi against prefoldin subunits is lethal when applied throughout development of the worm. RNAi targeting at later stages of development allows the observation of phenotypes in adult worms. The knockdown of *pfd-6* did not alter lifespan *per se*, but *pfd*-6 was partially necessary for the long-lived phenotype of a *daf-2* mutant of the insulin/insulin-like growth factor 1 (IGF-1) pathway (Son et al., 2018). Essentially, this effect was dependent on heat shock factor 1 (HSF-1) and forkhead box O (FOXO/DAF-16) transcription factors, which mediate lifespan extension in *daf-2* mutant animals. Moreover, the action of HSF-1 increased the expression of PFD-6 protein levels, possibly by activating the expression of *hsp-70* and *hsp-90*, which in turn bind and stabilize PFD-6. PFD-6 expression in the *daf-2* mutant was necessary for the upregulation of a subset of DAF-16 target genes in *daf-2* mutants. The role of PFD-6-dependent lifespan extension in *daf-2* mutants is likely related to its non-canonical function in the R2TP (Rvb1-Rvb2-Tah1-Pih1)/prefoldin-like complex rather than the canonical prefoldin complex (Son et al., 2018). This single study in *C. elegans* demonstrates that we are only in the incipient stages of understanding prefoldin function within a stress-loaded milieu, such as during aging. Much more work needs to be dedicated to unravelling stress-dependent functions of prefoldin.

## Implications of Prefoldin in Pathological Conditions

**FIGURE 5:**
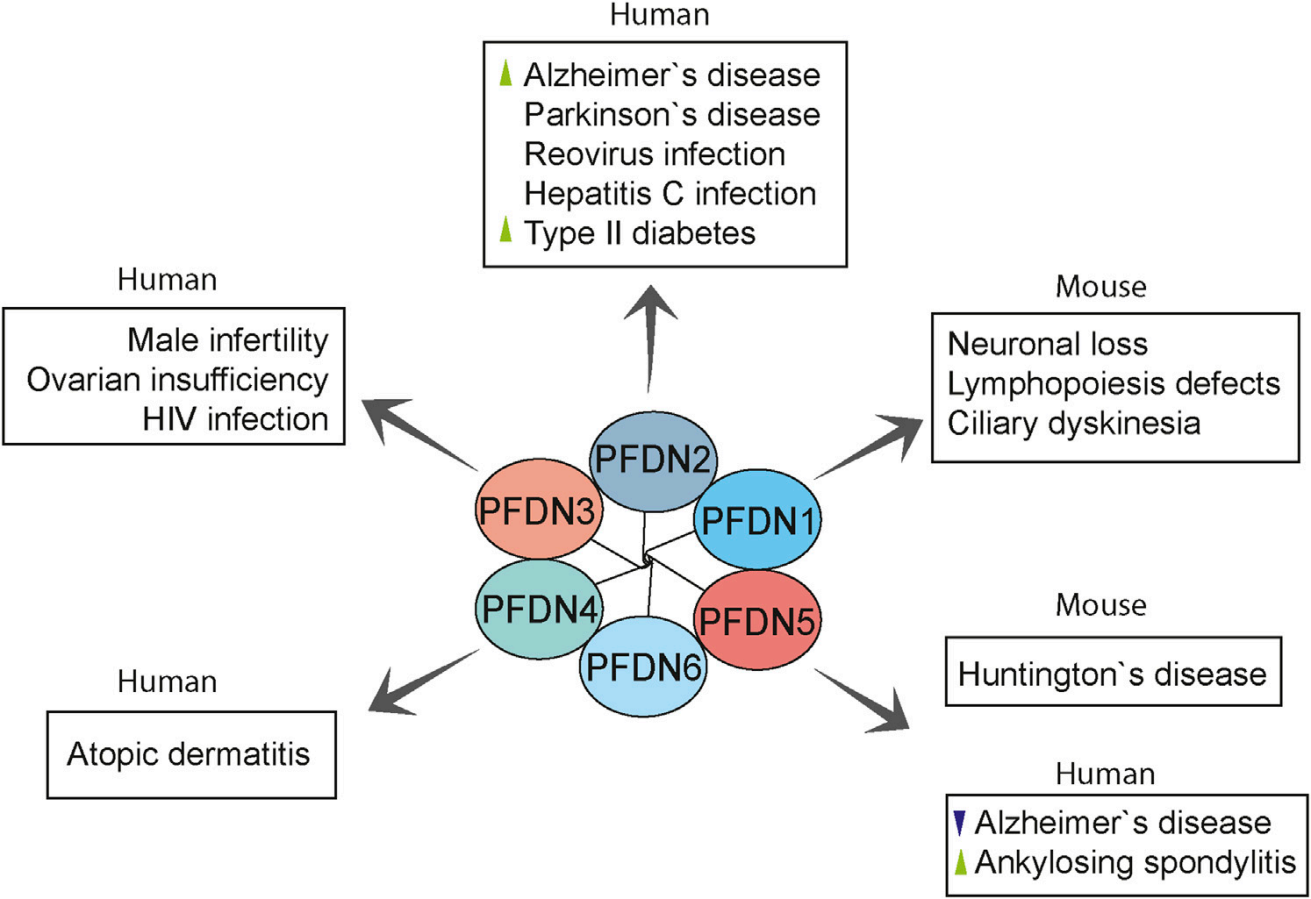
Overview of prefoldin-mediated diseases in humans and mouse models. Prefoldin subunits are involved in various pathologies other than cancer. PFDN2 and PFDN5 were linked with neurodegenerative diseases. PFDN2 and PFDN3 mediate some virus infections. Moreover, prefoldin subunits have distinct roles in the progression of rare anomalies. Changes in prefoldin subunit regulation in human diseases are indicated with arrowhead (green, upregulation; violet, downregulation).

Cytoskeletal proteins, such as actin and tubulin, are fundamental to some cellular processes, such as signal transduction, macromolecular trafficking, cell migration, and cell division (Small et al., 1999). Given the role of the prefoldin complex in the biogenesis of cytoskeletal proteins and its additional roles in the cell nucleus (Millan-Zambrano and Chavez, 2014), the prefoldin complex has been speculated to contribute to various disorders. The abnormal expression and function of the prefoldin complex are linked with various disorders, including neurodegenerative diseases, cancer, and viral infections (Figure 5 and Table 2).

**TABLE 2:** Altered expression of prefoldin subunits in human cancer.

Subunit	Pathogenic alteration	Type of cancer	References
All subunits	Upregulation	Gastric cancer	Yesseyeva et al. (2020)
PFDN1	Upregulation	Gastric cancer	Yesseyeva et al. (2020), Zhou et al. (2020)
	Upregulation	Lung cancer	Wang et al. (2017)
	Upregulation	Non-small-cell lung cancer	Peñate et al. (2020)
	Upregulation	Colorectal cancers	Wang P et al. (2015); Kwon et al. (2021)
PFDN2	Upregulation (gain of copy number)	Urothelial carcinoma	López et al. (2013), Riester et al. (2014)
		Bladder cancer	López et al. (2013)
		Gastric cancer	Yesseyeva et al. (2020)
	Genetic alterations	Breast tumors	Nami and Wang, (2018)
	Gain or loss of interaction with pathogenic or altered cellular proteins	Liver cancer	Chang et al., (2006); Dash et al., (2020); El-Serag, (2012); Farooq et al., (2013)
PFDN3 in intact PFDN complex	Downregulation	Von Hippel-Lindau syndrome (various types of cancers)	Chesnel et al., (2020); Kim et al., (2018); Tsuchiya et al., (1996)
PFDN4	Upregulation	Gastric cancer	Feng et al. (2009)
	Upregulation	Breast cancer	Collins et al. (2001)
	Upregulation	Hepatocellular carcinoma	Wang D et al., (2015)
	Downregulation	Colorectal cancer	Miyoshi et al. (2010)
PFDN5	Downregulation	Human leukemia, lymphoma, and tongue cancer	Fujioka et al. (2001)
	Upregulation of spliced variants	Thyroid neoplasms	Guimarães et al. (2006)
	Upregulation	Non-small cell lung cancer	Peñate et al. (2020)
	Downregulation	Parathyroid hyperplasia	Santamaría et al. (2005)
PFDN6	Downregulation	Pediatric acute lymphoblastic leukemia	Dehghan-Nayeri et al. (2017)

### Neurodegenerative Diseases

Neurodegenerative diseases are characterized by the progressive dysfunction of specific neurons and loss of their connections, mainly through the formation of long-insoluble protein fibrils and aggregates (Vaquer-Alicea and Diamond, 2019). Alzheimer’s disease is a major neurodegenerative disease that is characterized by progressive memory failure and age-related dementia, which are mainly caused by the aggregation of Aβ protein and formation of amyloid plaques in brain cells (Guo et al., 2020). Autopsies of human brain tissues and cDNA microarray analysis revealed the upregulation of PFDN2 transcript in Alzheimer’s disease brains compared with controls (Loring et al., 2001). The association between the prefoldin complex and Alzheimer’s disease was also confirmed by single-nucleotide polymorphism analysis and pathway assays (Broer et al., 2011). Interestingly, a study analyzing human whole blood mRNA expression data revealed downregulation of *PFDN5* in samples of Alzheimer’s patients, which could suggest a link to increased toxicity of Aβ (Tao et al., 2020). The prefoldin complex has also been shown to exert a protective effect against the pathogenesis of Huntington’s disease, a progressive neurodegenerative disorder, among a large family of proteopathies. Repeats of the CAG codon (which encodes glutamine) that are greater than 40 in exon 1 of the huntingtin gene elevate the risk of Huntington’s disease at age 65 (Langbehn et al., 2004; Langbehn et al., 2019). The extension of polyglutamine regions alters the length and oligomerization of pathogenic huntingtin that subsequently form β-sheet-rich fibrils and large toxic aggregates (Davies et al., 1997; DiFiglia et al., 1997). Unexpectedly, transgenic mice that carried an integrated human *HTT* gene with 300 CAG repeats (Q300) had a prolonged lifespan and longer disease onset compared with their parental mice with shorter CAG repeats (Q150). Transcription studies revealed that phenotype amelioration in Q300 mice was associated with the overexpression of *Pfdn5* and other genes that are involved in protein folding and localization (Tang et al., 2011). These genes could be therapeutic targets for improving symptoms of Huntington’s disease.

PFDN2 has been suggested to be involved in the pathogenesis of the most common neurodegenerative disease, Parkinson’s disease. miR-153–3 p microRNA downregulated α-synuclein, one protein that is associated with Parkinson’s disease, and decreased the abundance of PFDN2 in neuronal SH-SY5Y cells (Patil et al., 2015), suggesting a possible link between Parkinson’s disease and the prefoldin complex.

### Cancer

Cancer refers to a group of disorders that are caused by the uncontrolled division of cells that may start in every organ of the body and, in severe cases, spread to other organs (Hausman, 2019). Several studies reported the contribution of the prefoldin complex and its subunits to the onset and progression of some cancers (Table 2) (Liang et al., 2020; Herranz-Montoya et al., 2021). Higher prefoldin expression was observed in cancer cell lines compared with non-cancerous cells (Boudiaf-Benmammar et al., 2013). Additionally, all prefoldin subunits were found to be involved in the development of gastric cancer (Yesseyeva et al., 2020).

The role of cytoskeleton remodeling in cancer metastasis has been confirmed by numerous studies (Aseervatham, 2020). Therefore, one can speculate that prefoldin mediates cancer progression through its role in cytoskeletal proteostasis. Much evidence confirmed that high PFDN1 expression is associated with the metastasis and poor prognosis of gastric cancer, lung cancer, and colorectal cancer (Wang P. et al., 2015; Wang et al., 2017; Zhou et al., 2020; Kwon et al., 2021). High levels of PFDN1 activates the epithelial-mesenchymal transition (EMT), which is necessary for the initiation of metastasis. The mechanism by which PFDN1 initiates activation of the EMT is different in various tumors. For example, in gastric cancer cells, PFDN1 activates alterations of the EMT through Wnt/β-catenin signaling (Zhou et al., 2020). In lung tumors, PFDN1 contributes to growth and metastasis by suppressing the transcription of cyclin A. PFDN1 directly interacts with the cyclin A promoter at the transcription initiation site and inhibits cyclin A expression, which eventually activates the EMT and facilitates the migration of lung cancer cells (Wang et al., 2017). A study of PFDN1 mRNA and protein levels in clinical specimens from patients with colorectal cancer showed higher levels of PFDN1 in colorectal cancer samples, particularly the invasive form of the disease, compared with healthy tissues. PFDN1 promotes the proliferation and metastasis of colorectal cancer by maintaining cytoskeletal proteins, particularly F-actin and α-tubulin (Wang P. et al., 2015). Clinical specimens of non-small cell lung cancer revealed that the overexpression of PFDN1, PFDN3, and PFDN5 are significantly associated with higher mortality and metastasis in non-small cell lung cancer (Peñate et al., 2020). In contrast, microarray data revealed that *PFDN1* had stable expression levels in nasopharyngeal carcinoma samples, making it a suitable reference gene for expression studies (Guo et al., 2010).

PFDN1 functions as a tumor promoter, and its inhibition has a favorable effect in controlling the progression of some cancers. PFDN1 has been proposed as a target for the treatment of lung cancer (Wang et al., 2017). The silencing of PFDN1 decreased the expression of downstream proteins of Wnt/β-catenin signaling, suggesting that PFDN1 may be a specific target to attenuate the development of gastric cancer (Zhou et al., 2020). PFDN1 may also be a diagnostic biomarker and potential candidate for the treatment of colorectal cancer (Wang P. et al., 2015).

*PFDN2* is located on chromosome 1q23.3, and increases in its copy number are associated with a poor prognosis and treatment outcome of urothelial carcinoma (López et al., 2013; Riester et al., 2014). The amplification and overexpression of *PFDN2* were detected in high-grade tumors of the bladder. Additionally, high levels of PFDN2 were detected in urinary specimens from bladder cancer patients, suggesting that it may be a biomarker for prognosis, tumor staging, and clinical outcome (López et al., 2013). PFDN2 has been shown to interact with most α-tubulin and β-tubulin isoforms, and its genetic alterations are detected in 20% of breast tumors (Nami and Wang, 2018). Several studies indicated an association between PFDN2 and liver cancer. The alteration of one actin isoform, κ-actin, was discovered in some hepatocellular carcinoma tissues, with a decrease in its interaction with PFDN2. κ-Actin rearranges the actin cytoskeleton by replacing β-actin and becoming a dominant component of the actin cytoskeleton in cancerous hepatoma tissues (Chang et al., 2006). Interactions between F protein in hepatitis C virus with PFDN2 promote chronic infection (Tsao et al., 2006), which is known as a predominant risk factor for hepatocellular carcinoma (El-Serag, 2012; Dash et al., 2020). Additionally, alterations of histone deacetylase 1 protein expression were found in hepatocellular carcinoma, and this protein was shown to interact with prefoldin subunits in HepG2 human liver cancer cells (Farooq et al., 2013).

Low levels of the prefoldin complex, particularly the PFDN3 subunit, are associated with the severe phenotype of von Hippel-Lindau (VHL) syndrome (Chesnel et al., 2020). von Hippel-Lindau syndrome is a dominant cancer syndrome that predisposes to various familial or sporadic neoplasms of the central nervous system, adrenal glands, pancreas, retina, and kidney, including clear cell renal cell carcinoma. VHL syndrome is reviewed elsewhere (Varshney et al., 2017). von Hippel-Lindau syndrome is caused by mutations of the *VHL* tumor suppressor gene and the subsequent loss of its native protein product, pVHL. Tsuchiya and colleagues initially isolated PFDN3, also called von Hippel-Lindau binding protein 1 (VBP1), as a C-terminal binding partner of pVHL (Tsuchiya et al., 1996). PFDN3 was shown to stabilize pVHL and contribute to the pVHL-mediated degradation of hypoxia-inducible factor-1α, ultimately leading to the suppression of cancer metastasis (Kim et al., 2018). Although the main focus of the study by Kim and colleagues was on the PFDN3 subunit, recent research confirmed that the whole prefoldin complex is required to protect pVHL against aggregation. Lower levels of PFDN3 reduce stabilization of the whole prefoldin complex, eventually leading to the poor survival of clear cell renal cell carcinoma patients who harbor *VHL* missense mutations (Chesnel et al., 2020).

PFDN4, also known as protein C-1, was initially described as a transcription factor that regulates the cell cycle (Iijima et al., 1996). The expression of *PFDN4* is regulated by TWIST transcription factor, which plays an important role in the tumorigenesis and metastasis of gastric cancer. The silencing of TWIST increased *PFDN4* gene expression levels in gastric cancer cell lines, indicating that *PFDN4* might be related to gastric cancer (Feng et al., 2009). Accumulating evidence shows that the genetic locus of *PFDN4* confers potential susceptibility to breast cancer. The *PFDN4* gene is located on chromosome 20q13.2. A human genome sequence analysis of the 1.2 Mb amplified region of chromosome 20q13.2 showed that the *PFDN4* gene is located in the breast cancer amplicon. Amplification of this chromosome region increased *PFDN4* expression, suggesting that *PFDN4* is an oncogene that is associated with the development of breast cancer (Collins et al., 2001). However, quantitative polymerase chain reaction analysis of archival tissue samples from 54 patients with invasive ductal carcinoma of the breast did not show the significant overexpression of *PFDN4* in breast tumors (Born et al., 2005). These disparate results indicate the need for further studies to elucidate the role of *PFDN4* in the development of breast cancer.

The gain of whole chromosome 20q or specific regions therein also plays a role in progressive hepatocellular carcinoma. Analyses of copy number variations of chromosome 20q in hepatocellular carcinoma tumor samples and expression studies revealed that 20q13.12–13.33 gain correlates with the invasiveness of hepatocellular carcinoma. Moreover, *PFDN4* is among the genes whose higher expression is associated with a greater risk of extrahepatic metastasis and poor survival (Wang D. et al., 2015). Surgical specimens from 129 patients with colorectal cancer were assessed by Miyoshi et al. (2010) to determine *PFDN4* expression and its correlation with clinicopathological futures of colorectal cancer. Higher *PFDN4* expression was associated with better survival in colorectal cancer patients. This suggests that in the context of colorectal cancer, *PFDN4* functions as a tumor suppressor and may serve as a marker of favorable prognosis (Miyoshi et al., 2010). In breast and liver cancers, high *PFDN4* expression promoted carcinogenesis, suggesting that it is an oncogene (Collins et al., 2001; Wang D. et al., 2015). These findings indicate that the impact of *PFDN4* in the development of various cancers is tissue-specific.

PFDN5, also known as Myc modulator 1 (Mm-1), was shown to inhibit the transcriptional activity of c-MYC through various mechanisms (Mori et al., 1998; Yoshida et al., 2008). PFDN5 has been suggested to have a tumor-suppressing function. PFDN5 deficiency was shown to be associated with leukemia, lymphoma, and tongue cancer in humans and mammary cancer in canines (Fujioka et al., 2001; Hennecke et al., 2015).

The results of a cDNA microarray analysis of clinical colorectal cancer samples suggested that *PFDN5* may be a useful tumor histology and prognosis marker (Tsunoda et al., 2003). The high expression of an alternative splicing variant of *PFDN5* was detected in malignant thyroid neoplasms compared with benign lesions and normal thyroid tissues (Guimarães et al., 2006). Moreover, low *PFDN5* expression was found to correlate with advanced parathyroid hyperplasia (Santamaría et al., 2005).

The low protein and mRNA expression of PFDN6 was reported to be associated with resistance to the chemotherapy drug dexamethasone in pediatric acute lymphoblastic leukemia. Additionally, low mRNA levels of PFDN6 were detected in bone marrow samples from high-risk cases of pediatric acute lymphoblastic leukemia compared with the low risk and control healthy groups, suggesting that PFDN6 may be a predictive and treatment biomarker of pediatric acute lymphoblastic leukemia (Dehghan-Nayeri et al., 2017).

### Viral Infections

**FIGURE 6:**
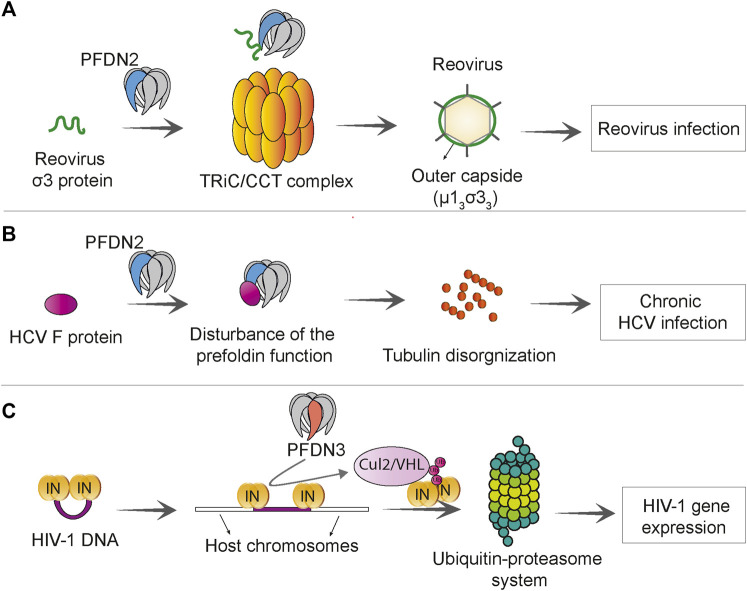
Prefoldin is linked to viral infections. **(A)** PFDN2 subunit of the prefoldin complex interacts with reovirus σ3 protein and increases the efficiency of TRiC/CCT -mediated μ1_3_σ3_3_ assembly, which promotes reovirus infection. **(B)** The Hepatitis C virus (HCV) F protein interacts with PFDN2 and disturbs the normal function of prefoldin complex in microtubule organization. This leads to persistence of virus in chronic HCV infection. **(C)** PFDN3 specifically interacts with HIV-1 integrase (IN) and mediates its interaction with cullin 2/VHL (Cul2/VHL) ubiquitin ligase, which subsequently leads to integrase “polyubiquitination” and proteasome-dependent degradation. This process promotes HIV-1 gene expression at a post-integration step.

Prefoldin subunits were found to promote some viral infections. The co-chaperone function of the prefoldin complex can be utilized to accelerate the folding of reovirus capsid protein (Knowlton et al., 2021). Reoviruses are rarely pathogenic in early human life but are associated with infection of the respiratory system (Jackson and Muldoon, 1973) and central nervous system (Tyler, 1998), gastroenteritis (Giordano et al., 2002), and celiac disease (Bouziat et al., 2017). The prefoldin complex was shown to augment the TRiC-mediated folding of σ3 reovirus capsid protein and enhance efficiency of σ3/μ1 assembly to form a heterohexameric proteinic capsid (μ1_3_σ3_3_). In this process, PFDN2 directly interacts with reovirus σ3 protein (Knowlton et al., 2021) (Figure 6A). Frameshift protein (F protein) of hepatitis C virus has been shown to bind to PFDN2 and impair function of the prefoldin complex. The F protein-PFDN2 interaction disturbs organization of the tubulin cytoskeleton, subsequently leading to the attenuation of hepatitis C virus replication and onset of chronic infection (Tsao et al., 2006) (Figure 6B).

PFDN3 has been shown to play a role in human immunodeficiency virus (HIV) infection. HIV-1 viral integrase is required for the integration of proviral DNA into the host genome, but after integration it remains bound to proviral DNA. PFDN3 was shown to bind to the viral integrase (Rain et al., 2009) and mediate its degradation through the ubiquitin-proteasome pathway, which facilitates the transcription of a viral gene. The prefoldin complex and cullin2/VHL ubiquitin ligase are required for appropriate expression of the HIV-1 gene and its post-integration replication (Mousnier et al., 2007) (Figure 6C).

### Autoimmune Response and Other Diseases

Clinical research has focused on the implications of prefoldin in tumor progression and as a diagnostic biomarker, but other medical conditions have also been related to prefoldin. Results of a mouse study showed that the loss of PFDN1 caused severe phenotypes that were related to cytoskeletal deficiencies, such as lymphopoiesis defects, neuronal loss, and ciliary dyskinesia (Cao et al., 2008). High plasma levels of PFDN2 antibody were reported in patients with type 2 diabetes, especially young adults, suggesting that it may be a clinical biomarker for this disease (Chang et al., 2017). PFDN3 is known to control the level of MutS homologue 4 (MSH4) protein by promoting its poly-ubiquitination in mitotic cells and subsequent degradation (Xu and Her, 2013). MSH4 is essential for meiotic recombination and proper gametogenesis. Lower expression levels of MSH4 were reported to be linked with human male infertility and primary ovarian insufficiency in women (Carlosama et al., 2017; Tang et al., 2020). A genome-wide association study found that the PFDN4 gene was a novel disease-susceptibility locus for atopic dermatitis, an inflammatory skin disorder, in the Japanese population (Hirota et al., 2012). To identify a diagnostic biomarker for uveitis in ankylosing spondylitis, an inflammatory disorder that usually affects the axial spine, Kwon *et al.* found higher serum levels of anti-PFDN5 antibody in ankylosing spondylitis patients with uveitis. The upregulation of PFDN5 was also confirmed in ocular lesions in a mouse model of this disorder, which was associated with high serum levels of anti-PFDN5 autoantibody. In uveitis, PFDN5 upregulation might be involved in protecting retinal cells against cell death. These observations indicate that serum levels of anti-PFDN5 antibody could be a predictive marker of the development of uveitis in ankylosing spondylitis patients (Kwon et al., 2019).

The PFD6 subunit of *Plasmodium falciparum* (*Pf*), a eukaryotic parasite that causes malaria, promotes its pathogenesis. Merozoite surface protein-1 (MSP-1) of *Pf* plays an essential role in the binding of merozoites to human erythrocytes and infecting erythrocytes. *Pf* PFD6 was shown to interact with MSP-1 suggesting that *Pf* PFD6 might stabilize and assist the trafficking of MSP-1, which indirectly promotes erythrocyte invasion and pathogenicity of malaria (Kumar et al., 2020).

## Prefoldin as a Promising Candidate in Nanomedicine

Several studies investigated the ability of the prefoldin complex to serve as a nanorobot for the intelligent delivery of nano cargo, such as nano drugs. The special structure of prefoldin, which consists of six long coiled-coil tentacles with a flexible central cavity, and the fact that it does not need to utilize ATP to capture and deliver substrates make it an attractive candidate for designing a nano actuator. By introducing amino acid substitutions, a novel prefoldin nano actuator (PNA) was designed that could manipulate hydrophobic nano cargo and inhibit the formation of pathogenic Aβ oligomers. The latter ability suggests that the designed PNA can be effective for the treatment of Alzheimer’s disease (Sacks et al., 2018). The binding and release of nano cargo to the PNA cavity can be controlled by pH and temperature. High temperature decreases the area of the central cavity without affecting the overall conformation of prefoldin. The central cavity of PNA is able to capture a positively charged nano cargo at neutral pH and release it at acidic pH (Ghaffari et al., 2012; Shokuhfar et al., 2012). Given the higher temperature and lower pH of the tumor microenvironment compared with normal tissues (Zhang W. et al., 2020), utilization of the PNA may be a site-specific therapeutic strategy whereby an anti-tumor drug is released only at the tumor site while other normal tissues remain unaffected.

## Perspectives

The molecular co-chaperone prefoldin is a multifaceted protein complex, but the molecular pathways to which it is linked remain poorly described. The diverse involvement of prefoldin in various interconnected cellular functions, ranging from transcriptional regulation to co-translational holdase and post-translational degradation processes, hamper rapid progress in this research field. One future research direction will be to elucidate the cellular and environmental conditions that allow the prefoldin complex to switch between its various functions. Understanding the involvement and regulation of prefoldin under both acute and chronic stress conditions requires further effort. Accumulating evidence suggests that specific prefoldin subunits have functions that are separate from the canonical complex. It is unclear how the balance between formation of the complex and functions of single subunits is regulated. Proteomics approaches that compare landscapes of prefoldin subunit interactions with each other could shed light on independent subunit functions. Pleiotropic positive and negative genetic interactions that are revealed in large-scale screens, mostly in yeast, suggest the existence of extended prefoldin interactors. More efforts should be made to determine whether prefoldin can fulfil its functions without its downstream chaperone TRiC/CCT. Other chaperones could also function together with prefoldin or direct the role of prefoldin under certain cellular conditions.

Finally, the prefoldin complex and its subunits appear to be promising for the discovery of biomarkers for human diseases, including various cancers. More basic research is needed to understand the cause of alterations of prefoldin regulation under pathological conditions to verify whether prefoldin can be a target for treating these diseases.
